# Correction of lower limb deformities with fixator assisted nailing

**DOI:** 10.1016/j.amsu.2019.07.006

**Published:** 2019-07-08

**Authors:** Hussain Wahab, Shah Fahad, Talal Aqueel Noor-us-Sabah, Yasir Mohib, Haroon Ur Rashid, Masood Umer

**Affiliations:** Section of Orthopedics, Department of Surgery, The Aga Khan University Hospital, Karachi, Pakistan

**Keywords:** Deformity correction, External fixator, Fixator-assisted nailing, Intramedullary nail

## Abstract

**Introduction:**

Deformities of the lower extremities can be congenital or acquired. Various surgical treatments have been employed for such disorders including osteotomy followed by either external fixation, internal fixation or external fixator assisted internal fixation. The aim of surgery is correction of deformity and restoration of mechanical axis and joint line. External fixator assisted internal fixation with intramedullary (IM) nail insertion is considered the gold standard, however, it is less commonly practiced as expertise required are usually not available at most centers. This study was conducted to assess the radiological and functional outcomes after fixator assisted IM nailing for correction of lower limbs deformity.

**Methods:**

It was a retrospective study at a tertiary care hospital. All cases of lower limb deformity whose correction was done with fixator assisted IM nailing from 2010 till 2017 were analyzed. Pre Op x-rays and post op x rays were analyzed for Mechanical Axis Deviation (MAD), anatomical Lateral Distal Femoral Angle (aLDFA), mechanical Lateral Distal Femoral Angle (mLDFA) and Medial Proximal Tibial Angle (MPTA), post-operative activity and functional status of the patients. Data was analyzed using SPSS.

**Results:**

Thirteen patients were included in the study. Fixator assisted IM nailing was performed on 29 long bones of these patients including 16 femur and 13 tibial deformities. Pre Op and Post Op comparison was done for MAD, aLDFA, mLDFA, MPTA. Pre op mean MAD was 38.87 ± 25.58 post op mean MAD 17.54 ± 12.25 mm. Only 2 of our patients developed knee stiffness for which manipulation under anesthesia was done. One of our patients developed weakness in toe extension, which recovered after 6 months. On follow up evaluation patients had normal range of motion and no functional limitation.

**Conclusion:**

Fixator assisted IM nailing for deformity correction is a better option, because it has advantages of both external fixator and internal fixator. Knee stiffness associated with external fixator can be prevented. It is more convenient for patient.

## Highlights

• Deformity correction is a practical challenge, even in most experienced hands.
• The aim of surgery is correction of deformity.
• The Fixator assisted nailing is gold standard methods of deformity correction.

## 1 Introduction

Deformity correction is a practical challenge, even in most experienced hands. There are various conditions that may cause lower limb deformities. A few include metabolic bone disease (including hypophosphataemic rickets, hypophospatasia, and renal osteodystrophy), congenital deformities around the knee joint, acquired deformities secondary to trauma leading to malunion, or acquired hypertrophic nonunion or as a result of the poliomyelitis. Osteotomies and correction of deformity followed by fixation (internal, external or both) is the mainstay of treatment, once skeletal maturity is achieved. Regardless of technique, the goals of correction include restoration of mechanical axis, joint line restoration and correction of deformity in all plane [1].

Deformity of the lower limb leads to early osteoarthritis of the weight bearing joint [2]. There are different ways for correction of the limb deformity. Two main options are; external fixation and internal fixation [1], each with its advantages and disadvantages. The Fixator assisted nailing is considered the gold standard, because it has advantages of both methods of deformity correction [3]. First described by Paley in 1997, the internal fixation (i.e.; intramedullary nail) gives stability, patient convenience and prevents recurrence of the deformity; the temporary external fixator helps in accuracy and adjustability [4].

In this study we present our experience of deformity correction of lower limb with fixator assisted intramedullary nailing, its radiological outcome and complications.

## 2 Material and methods

This is a retrospective analysis of all thirteen patients who underwent deformity correction of the lower limb with fixator assisted intramedullary nailing from January to 2010 till June 2017 in our tertiary care hospital. Approval was taken from hospital's ethical review committee. All those patients who underwent fixator assisted intramedullary nailing of tibia or femurs for deformity correction were included. Patients who lost to follow up and those with missing records were excluded from the study. All surgeries were performed by a senior surgeon in orthopedics with experience in deformity correction surgeries. The work has been reported in line with the PROCESS criteria [5].

## 3 Pre-operative planning

On presentation detailed history was taken and patients were examined for range of motion at knee, hip and ankle. Varus, valgus and rotational malalignment and neurological assessment of lower limb was done. A full length weight bearing radiograph was obtained, including hip, knee and ankle joint shown in Fig. 1. Mechanical axis deviations (MAD), anatomical Lateral Distal Femoral Angle (aLDFA), mechanical Lateral Distal Femoral Angle (mLDFA), Medial proximal tibial angle (MPTA) were calculated from weight bearing radiographs. Centre of rotation of angulation was identified, and paper tracing of the radiograph was obtained. CORA (center of rotation of angulation) was obtained on paper tracing and cuts were made and paper tracing was reorganized.

**Fig. 1:**
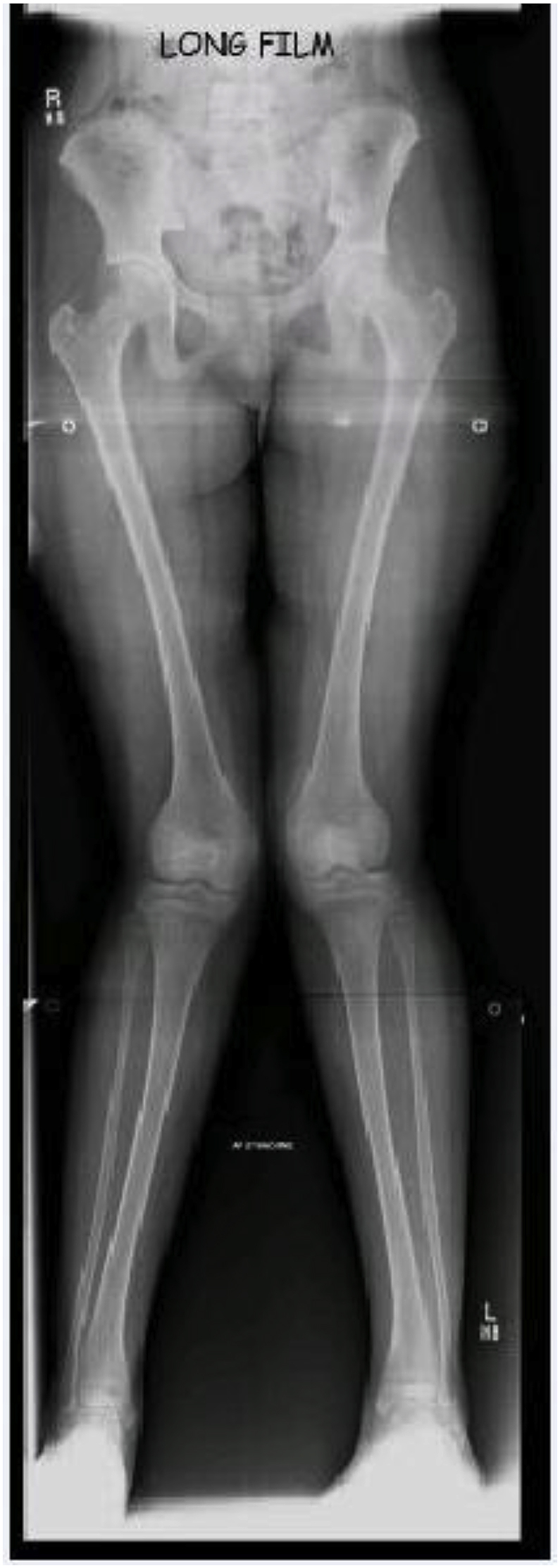
Sacnogram (full length weight bearing x ray of hip, knee and ankle joint).

Nail diameter, and length was also measured pre operatively, location of poller screw (interference screws) were also identified.

## 4 Intra operative

Intra operative patient was kept supine position, after prep and drape. Two pairs of schanz screws were passed 1 pair proximal and 1 distal part of the tibia/femur. Per cutaneous osteotomy was performed as per preoperative plan. Using schanz screw as joystick, translation is done and correction of deformity as per pre-operative planning.

Once the desired correction was achieved by the external fixator, poller screws were passed to guide the guide wire in the planned direction and then reaming was followed. Intramedullary nail was passed and locked with static screws proximally and distally (Fig. 2), and external fixator was then removed. For distal femoral deformity, tibial intramedullary nails were used in retrograde position. Initially schanz screws were passed in posterior part of the tibia to provide enough space for intramedullary nail as shown in Fig. 3.

**Fig. 2:**
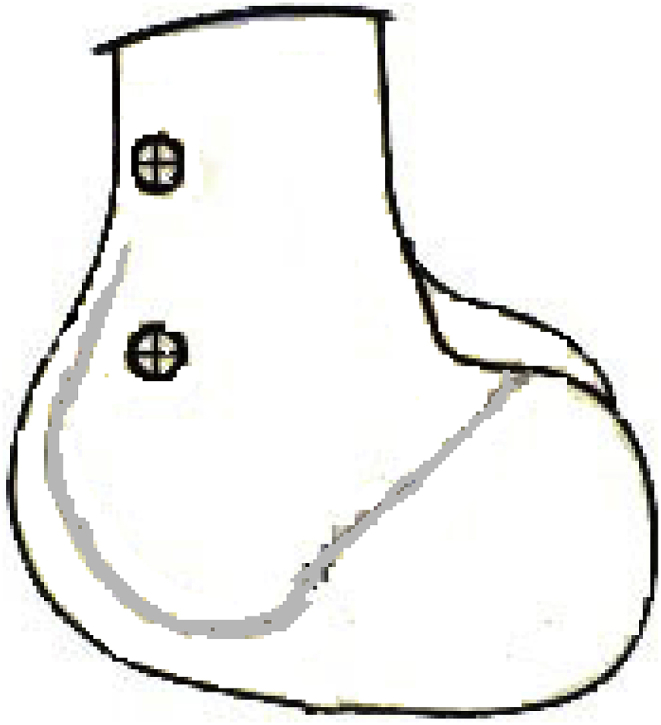
Showing position of schanz screws in distal femur.

**Fig. 3:**
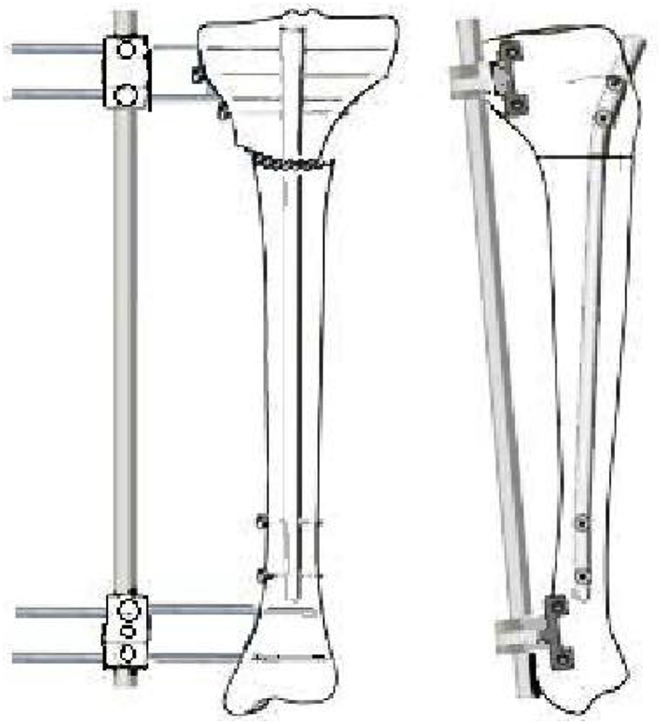
Translation at osteotomy site with external fixator applied and intramedullary nail in place.

On operation table cautery test was performed to check the alignment of the lower limb and assess the correction of deformity.

## 5 Post-operative

Post operatively patient was closely observed for compartment syndrome and nerve injury. Post-operatively patient was ambulated full weight bearing with walker, and a full length weight bearing radiograph is obtained once patient was stabilized. Mechanical axis deviation (MAD), anatomical Lateral Distal Femoral Angle (aLDFA), mechanical Lateral Distal Femoral Angle (mLDFA), Medial proximal tibial angle (MPTA) were calculated from weight bearing post op radiographs(Fig. 4). Patients were followed at 2 weeks, 6 weeks and 6 months and one year. Patients were assessed for union at osteotomy, recurrence of deformity clinically and on plain radiographs on every visit.

**Fig. 4:**
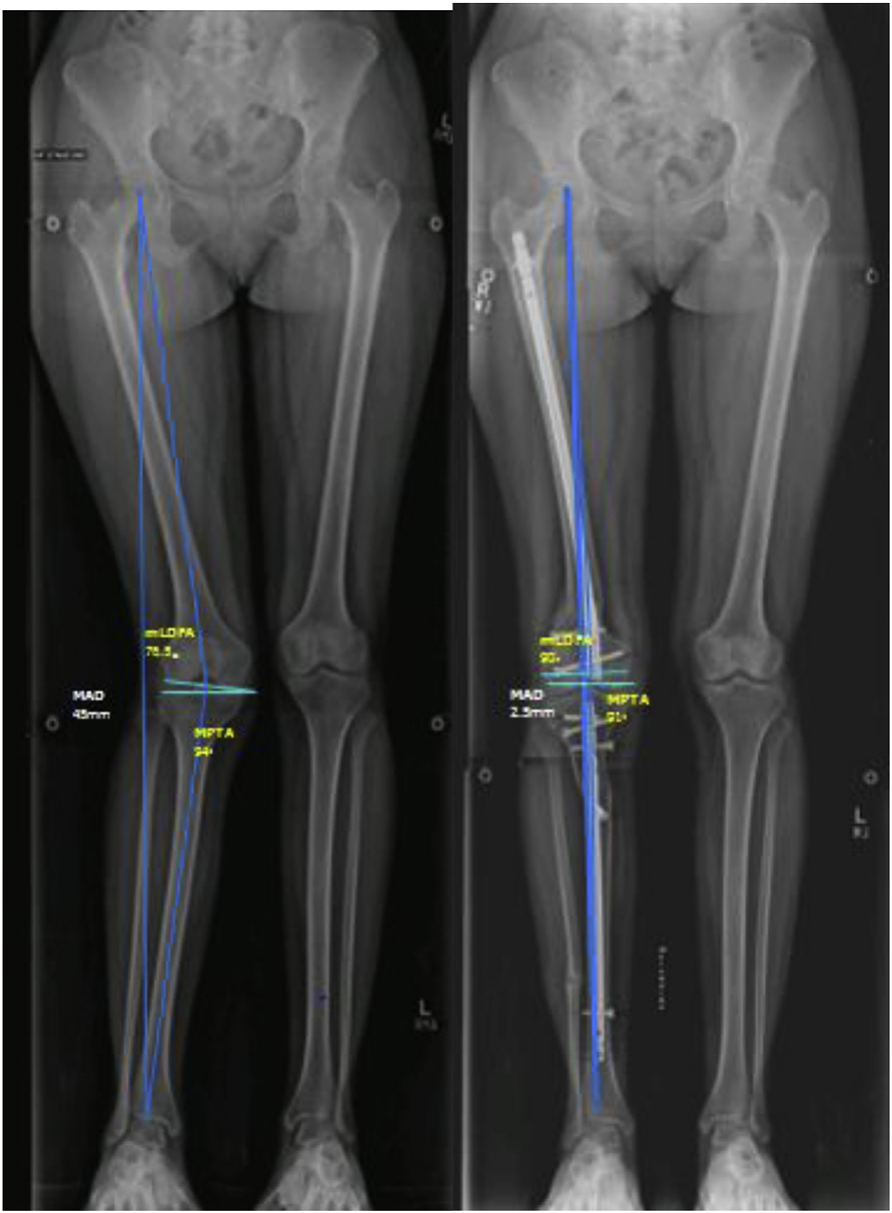
A. Preoperative radiographs of 15 years old girl with genu valgum deformity at right knee. Mechanical axis deviation (MAD) is 45mm, mechanical Distal Femoral Angle(mLDFA) is 76.5°, Medial Tibial Angle(MPTA) is 94°. B. postoperative radiographs after fixator assisted nail correction of deformity. Mechanical axis deviation (MAD) is 2.5mm, mechanical Distal Femoral Angle(mLDFA) is 90°, Medial Tibial Angle(MPTA) is 91.

## 6 Results

Total number of patients who underwent deformity correction of long bones of lower limb with fixator assisted nailing was 13, 7 were male and 6 were female. Age range was from 14 to 46 years, mean age was 22.57 years. Causes of the deformity were idiopathic deformity in 4 cases, post traumatic deformity in 2 cases, osteogenesis imperfect in 3 cases, 2 cases of hypophosphataemic rickets, 1 case had osteochondroma and 1 case was having renal tubular acidosis as shown in Table 1.

**Table 1:** Table showing the diagnosis of the 23 patients with deformity at lower limb and the bone involved.

Number	Diagnosis	Deformity	Bone segment involved
1	Idiopathic	Genu valgum	Femur
2	Idiopathic	Genu valgum	Femur
3	Post trauma deformity	Genu varus	Femur
4	Hypophosphatemic rickets	Genu valgum	Tibia + Femur
5	Hypophosphatemic rickets	Genu valgum	Tibia + Femur
6	Osteochondroma	Genu valgus	Tibia
7		Genu valgum	Tibia + Femur
8	Hypophosphatemic Rickets	Genu valgus + ro curvatum	Tibia
9	Idiopathic	Genu valgum	Tibia
10	Idiopathic	Genu valgum	Tibia
11	Rickets	Genu valgum	Femur
12	Idiopathic	Genu valgum	Femur
13	Renal tubular acidosis	Genu valgum	Femur
14	Renal tubular acidosis	Genu valgum	Femur
15	Osteogenesis imperfect	Varus and pro curvatum	Tibia
16	Osteogenesis imperfect	Varus and pro curvatum	Tibia
17	Osteogenesis imperfect	Tibia: Varus + pro curvatum Femur: Genu varus	Tibia + Femur
18	Osteogenesis imperfect	Tibia: Genu varus + pro curvatum Femur: Genu varus	Tibia + Femur
19	Post traumatic deformity	Genu valgus	Tibia
20	Idiopathic	Genu valgum	Femur
21	Idiopathic	Genu valgum	Femur

Fixator assisted nailing done was for 16 femurs, and 13 tibias. These were further subdivided in sub groups of bilateral femur (4 = 8 Femurs), bilateral tibia (3 = 6 tibia) and bilateral Tibia and Femur (3 = 6 femur and 6 tibia), only isolated femur (2 = 2) only isolated tibia (1 = 1 tibia) as shown in Table 1. On follow up visits, recurrence of deformity, range of motion, knee stiffness and neurological assessment was done.

Two of our patients developed knee stiffness for which manipulation under anesthesia was done. One of them is still having stiffness range of motion 0–90° and using stick for walking. One patient developed weakness in toes extension after fibular osteotomy, he recovered within 6 months. Rest of patients had normal range of motion and no functional limitation.

Pre Op and Post Op comparison was done for mechanical axis deviation (MAD), anatomical Lateral Distal Femoral Angle (aLDFA), mechanical Lateral Distal Femoral Angle (mLDFA), and Medial Proximal Tibial Angle (MPTA). The mean mechanical axis deviation of the lower extremity improved from 38.87 mm before the surgery to 17.54mm after the surgery. The mean anatomical Lateral Distal Femoral Angle (aLDFA) improved from 73.16 ± 8.38° before the surgery to 79.13 ± 5.03° after the surgery, the mean mechanical Lateral Distal Femoral Angle (mLDFA) improved from 78.20 ± 8.34° before the surgery to 85.03 ± 5.17° after the surgery, the mean Medial Proximal Tibial Angle (MPTA) improved from 92.80 ± 8.31° before the surgery to 89.92 ± 2.57° post operatively.

## 7 Discussion

Deformity of the lower limb leads to early osteoarthritis of the weight bearing joint and psychological and social disability. The aim of deformity correction to restore joint orientation angles within normal anatomical range to prevent the developmental of early osteoarthritis [6]. Of many different methods for correction of the deformity are External fixation and internal fixation. External fixation is less invasive and it is more accurate to correct the deformity and can also be readjusted, but for is cumbersome for the patient [3]. Post-operative complications of external fixator include pin tracts infection and joint stiffness [2,3]. Internal fixation gives stability and patient's comfort increases with it, but it lacks accuracy, adjustability and is technically demanding [3].

Fixator assisted nailing is considered the gold standard, because it has advantages of both internal and external fixation for deformity correction and avoid the disadvantages of these methods of fixation [3]. The internal fixation (i.e.; intramedullary nail), gives stability, patient convenience and prevent recurrence of the deformity, the temporary external fixator helps in accuracy and adjustability. The results of the fixator assisted nailing for deformity correction is comparable to the deformity correction with Ilizarov [7].

In our study post correction most of the angles were restored, mean was with in the normal range. The normal range of aLDFA (79–83), mLDFA (85–90), MPTA(85 to 90) and MAD (0–15 mm) [8,9].

*Hans Michael* et al. conducted a study in 2006, he compared Taylor Spatial Frame with the Ilizarov Ring fixator with regard to accuracy of deformity of correction. 90.7% correction was achieved in the Taylor Spatial Frame group, and 55.7% in Ilizarov ring fixator group [10].

HR song et al. evaluated the outcomes of deformity correction using different modalities for correction rickets. Out of 55 long bones analyzed, in 28 distraction osteogenesis and in 27 acute corrections was done. External fixator was used in 24 long bones, and IM nail was used in 6 long bones and external fixation with IM nail was used in 25 long bones. In his results complications related to different modality of correction were, nail related 3 of 6 with IM nailing, 2 of 25 with external fixator and nailing. Recurrence of deformity or re-fracture associated with distraction osteogenesis by external fixator alone was 10 of 21, with IM nailing was 4 of 6 and 1of 25 with acute correction with external fixator and IM nailing. He recommended use of IM nail and external fixator for correction of deformity secondary to hypophosphatemic rickets [11] **.**

In our study external fixator was removed on the operative table after achieving desired correction of deformity and fixation with intramedullary screw. There was no recurrence of deformity. Complications in our study were post-surgery knee stiff ness in two cases and peroneal nerve injury in 1 case. 1 patient was 17 years old boy who underwent for deformity correction of femur secondary to trauma, post op he developed knee stiffness, on 3 years follow up that patient still has limited range of motion of knee joint 0–90°, and using stick for walking. Immediate correction of deformity jeopardize neurovascular structures especially peroneal nerve after correction of valgus knee [12]. Second patient was 16 years old girl with genu valgum, she developed post-operative knee stiffness, manipulation under anesthesia was done and she regained normal range of motion on subsequent follow ups. Third patient was 28 years old boy with valgus deformity of tibia secondary to trauma. He developed weakness in toes extension secondary to nerve injury after fibular osteotomy; he recovered within 6 month of the surgery.

Limitations our study are, small number of cases and retrospective study.

## Conflict of interests and funds

No conflict of interest. No funding from any source for this project.

## Ethical approval

All procedures performed in studies involving human participants were in accordance with the ethical standards of the institutional and/or national research committee and with the 1964 Helsinki declaration and its later amendments or comparable ethical standards. As this is a retrospective study formal consent is not required.

## 8 Conclusion

Fixator assisted IM nailing for deformity correction is a better option, because it has advantages of both external fixator and internal fixator. It is more convenient for patients, patients can be ambulated full weight bearing early, and complications of knee stiffness associated with external fixator/Ilizarov can be prevented. Chances of recurrence of deformity and re-fracture can also be prevented.

## Provenance and peer review

Not commissioned, internally reviewed.

## Ethical approval

Hospital ethical review committee approval was taken.

## Sources of funding

None.

## Author contribution

Hussain Wahab: first proposal, data collection, analysis and manuscript writing and editing.

Shah Fahad:data collection, analysis and manuscript writing and editing.

Talal Aqeel: data analysis and manuscript writing and editing.

Noorus Sabah:Data collection, analysis and manuscript writing.

Yasir Mohib: review and editing.

Masood Umer: review and editing.

Haroon Rashid: review and editing.

## Conflicts of interest

No conflict of interest.

## Trial registry number

NCT03847064.

## Guarantor

Haroon Rashid.

Talal Aqeel.

## Appendix A Supplementary data

Process Checklist Process Checklist

DataProfile DataProfile

## References

[1] Eralp L, Kocaoğlu M, Çakmak M, Ozden V. reportA Correction of Windswept Deformity by Fixator Assisted Nailing - A Report of Two Cases 2004. 1065-1068 p. 10.1302/0301-620x.86b7.14923 15446540

[2] Sharma L., Song J., Felson D.T., Cahue S., Shamiyeh E., Dunlop D.D. (2001). The role of knee alignment in disease progression and functional decline in knee osteoarthritis. J. Am. Med. Assoc..

[3] Kocaoglu M., Bilen F.E. (2011). Fixator-assisted nailing for correction of long bone deformities. Operat. Tech. Orthop..

[4] Paley D., Herzenberg J.E., Paremain G., Bhave A. (1997). Femoral lengthening over an intramedullary nail. A matched-case comparison with Ilizarov femoral lengthening. J Bone Joint Surg Am.

[5] Agha R.A., Borrelli M.R., Farwana R., Koshy K., Fowler A.J., Orgill D.P. (2018). The PROCESS 2018 statement: updating consensus preferred reporting of CasE series in surgery (PROCESS) guidelines. Int. J. Surg..

[6] Pelletier J.-P., Raynauld J.-P., Berthiaume M.-J., Abram F., Choquette D., Haraoui B. (2007). Risk factors associated with the loss of cartilage volume on weight-bearing areas in knee osteoarthritis patients assessed by quantitative magnetic resonance imaging: a longitudinal study. Arthritis Res. Ther..

[7] Eralp L., Kocaoglu M., Toker B., Balcı H.I., Awad A. (2011). Comparison of fixator-assisted nailing versus circular external fixator for bone realignment of lower extremity angular deformities in rickets disease. Arch. Orthop. Trauma. Surg..

[8] Fawdington R.A., Johnson B., Kiely N.T. (2014). Lower limb deformity assessment and correction. Orthop. Traumatol..

[9] Paley D., Tetsworth K. (1992). Mechanical axis deviation of the lower limbs. Preoperative planning of uniapical angular deformities of the tibia or femur. Clin. Orthop. Relat. Res..

[10] Manner H.M., Huebl M., Radler C., Ganger R., Petje G., Grill F. (2007). Accuracy of complex lower-limb deformity correction with external fixation: a comparison of the Taylor Spatial Frame with the Ilizarov ring fixator. J Child Orthop.

[11] Song H.-R., Raju S.V., Kumar S., Lee S.-H., Suh S.-W., Kim J.-R. (2006). Deformity correction by external fixation and/or intramedullary nailing in hypophosphatemic rickets. Acta Orthop..

[12] Paley D., Herzenberg J.E., Bor N. (1997). Fixator-assisted nailing of femoral and tibial deformities. Tech. Orthop..

